# Temporal Quantitative Phosphoproteomics Profiling of Interleukin-33 Signaling Network Reveals Unique Modulators of Monocyte Activation

**DOI:** 10.3390/cells11010138

**Published:** 2022-01-01

**Authors:** Devasahayam Arokia Balaya Rex, Yashwanth Subbannayya, Prashant Kumar Modi, Akhina Palollathil, Lathika Gopalakrishnan, Yashodhar P. Bhandary, Thottethodi Subrahmanya Keshava Prasad, Sneha M. Pinto

**Affiliations:** 1 Center for Systems Biology and Molecular Medicine, Yenepoya Research Centre, Yenepoya (Deemed to be University), Mangalore 575018, India; rexprem@yenepoya.edu.in (D.A.B.R.); prashantmodi@yenepoya.edu.in (P.K.M.); akhinap@yenepoya.edu.in (A.P.); 2 Centre of Molecular Inflammation Research (CEMIR), Department of Clinical and Molecular Medicine (IKOM), Norwegian University of Science and Technology, N-7491 Trondheim, Norway; 3 Institute of Bioinformatics, International Tech Park, Bangalore 560066, India; lathika.gnair@gmail.com; 4 Manipal Academy of Higher Education (MAHE), Manipal 576104, India; 5 Yenepoya Research Centre, Yenepoya (Deemed to be University), Mangalore 575018, India; yash28bhandary@gmail.com

**Keywords:** IL-33, cytokine signaling, phosphoproteomics, mass spectrometry, DNA damage, DNA repair, inflammation

## Abstract

Interleukin-33 (IL-33), a member of the IL-1 superfamily cytokines, is an endogenous danger signal and a nuclear-associated cytokine. It is one of the essential mediators of both innate and adaptive immune responses. Aberrant IL-33 signaling has been demonstrated to play a defensive role against various infectious and inflammatory diseases. Although the signaling responses mediated by IL-33 have been previously reported, the temporal signaling dynamics are yet to be explored. To this end, we applied quantitative temporal phosphoproteomics analysis to elucidate pathways and proteins induced by IL-33 in THP-1 monocytes. Employing a TMT labeling-based quantitation and titanium dioxide (TiO_2_)-based phosphopeptide enrichment strategy followed by mass spectrometry analysis, we identified and quantified 9448 unique phosphopeptides corresponding to 3392 proteins that showed differential regulation. Of these, 171 protein kinases, 60 phosphatases and 178 transcription factors were regulated at different phases of IL-33 signaling. In addition to the confirmed activation of canonical signaling modules including MAPK, NFκB, PI3K/AKT modules, pathway analysis of the time-dependent phosphorylation dynamics revealed enrichment of several cellular processes, including leukocyte adhesion, response to reactive oxygen species, cell cycle checkpoints, DNA damage and repair pathways. The detailed quantitative phosphoproteomic map of IL-33 signaling will serve as a potentially useful resource to study its function in the context of inflammatory and pathological conditions.

## 1. Introduction

Interleukin-33 (IL-33), a member of the IL-1 family of cytokines, is an endogenous danger signal and a nuclear-associated cytokine. IL-33 is primarily released from epithelial cells and fibroblasts following cell injury to alert the immune system of tissue damage during trauma or infection [1]. It is one of the essential mediators of both innate and adaptive immune responses, playing a vital role against a gamut of infectious and inflammatory diseases [2,3,4].

Exogenous IL-33 is largely known to initiate a Type 2 immune response by binding to a heterodimeric complex consisting of the ST2 receptor and a co-receptor- IL 1 receptor accessory protein (IL 1RAcP) expressed in hematopoietic cells, such as T-helper type 2 (Th2) cells, mast cells macrophages, and eosinophils, among others [5,6]. This complex leads to a cascade of downstream signaling events, including the recruitment of adaptor proteins, such as MYD88, IL 1R associated kinase 1 (IRAK1), and IRAK4, resulting in the activation of mitogen-activated protein kinases (MAPKs) and nuclear factor κB (NFκB) through tumor necrosis factor (TNF) receptor-associated factor 6 (TRAF6) [7]. The signaling cascade culminates in the regulation of expression and release of Th2 cytokines as well as other pro-inflammatory cytokines, such as IL-8, and IL-6, from the cells involved in innate immune signaling [8,9,10,11,12]. Increasing evidence now demonstrates that IL-33 also promotes Type 1 immune response, regulatory T cell responses and ILC2 activation [13]. As a nuclear alarmin, the nuclear localization or retention of IL-33 is vital for immune homeostasis by limiting the potent pro-inflammatory effects of IL-33 [14]. Aberrant IL-33 signaling is therefore widely implicated in the pathogenesis of Th2-related diseases, such as acute and chronic inflammatory diseases, including asthma, atopic dermatitis, ulcerative colitis and rheumatoid arthritis, among others [7].

Despite our knowledge of signaling mediated by IL-33, the transitional signaling events culminating in the activation of cellular processes and networks that regulate the production of pro-inflammatory cytokines in varied cell types remains poorly understood [7]. Although IL-33 signaling dynamics using a quantitative proteomic approach was described earlier, it was performed at a single time point in mouse macrophage cell line (RAW264.7) [15]. Moreover, its role in human monocyte signaling is not well studied. Therefore, an in-depth understanding of IL-33-induced phosphorylation dynamics is imperative as it will enable the identification of critical hubs and signaling nodes regulating immune cell function, secretion and other cellular responses and provide leads on novel molecular targets for treatment strategies to resolve inflammation.

To this end, we employed a quantitative multiplexed phosphoproteomics approach to elucidate the temporal signaling mediated by IL-33 in human monocytic cells. In addition to identifying the temporal dynamics of canonical MAPK/NFκB signaling pathways, our results reveal the unique dynamic phosphoproteome profiles of cellular processes, including that of DNA damage response, enriched biological functions associated with DNA repair, response to reactive oxygen species, cell cycle checkpoints and mRNA splicing, all key modulators of monocytic immune response and mediators of gene expression.

## 2. Materials and Methods

### 2.1. Cell Culture and Sample Preparation

THP-1 cells (ATCC®) were maintained in RPMI 1640 media (HiMedia) supplemented with 10% (*v*/*v*) FBS and 1% (*v*/*v*) antibiotic- antimycotic solution (Thermo Fisher Scientific, Cat# 15240062) at a density of 2 × 10^5^ cells/mL in a humidified incubator at 37 °C with 5.0% CO_2_ [16]. The cells were stimulated with 50 ng/mL human rIL-33 (R&D Systems) for different time intervals (i.e., 0, 5, 10, 15, 30, 40, 60, 120 and 240 min). The 50 ng/mL concentration was based on previous studies [17,18,19,20]. All the treatments were performed in triplicate. The cell pellets were washed with 1% PBS and lysed with lysis buffer (4% sodium dodecyl sulfate (SDS) in 50 mM triethylammonium bicarbonate (TEABC), sodium orthovanadate (1 mM), sodium pyrophosphatase (2.5 mM), and beta-glycerophosphate (1 mM)) and heated at 95 °C on a dry bath for 10 min, followed by centrifugation at 12,000× *g* for 20 min. Proteins were extracted by probe sonication using Q-Sonica (Cole-Parmer, Mumbai, India) and the concentration was estimated using Bicinchoninic acid assay (BCA) (Pierce, Waltham, MA, USA).

### 2.2. Treatment of Cells with the MEK Inhibitor U0126

THP-1 cells (2 × 10^5^ cells/mL) were pre-treated with 10 μM U0126 (1,4-diamino-2,3-dicyano-1,4-bis [2-aminophenylthio] butadiene (Sigma Aldrich, Bangalore, India) for 30 min before stimulation with IL-33 at the indicated timepoints. The cells were washed with 1% PBS and lysed using SDS lysis buffer for protein extraction after stimulation.

### 2.3. Western Blot for MAPK Signaling Pathway

A total of 20 ug from cell lysates was separated on 10–12% sodium dodecyl sulphate-polyacrylamide gel electrophoresis (SDS-PAGE) and transferred onto the nitrocellulose membranes (BioRad, Hercules, CA, USA) using a wet transfer method. The membranes were blocked with 5% non-fat milk prepared in phosphate-buffered saline containing Tween 20 (PBS-T) for 1 h at room temperature followed by incubation with primary antibodies overnight at 4 °C under gentle agitation. The primary antibodies probed included: anti-ERK (9107S), anti-phospho-ERK (4370S), anti-Phospho-IκBα (9246S), anti-NFκB (8242T), anti-phospho-NFκB (3033T) purchased from Cell Signaling Technology (all diluted at 1:1000) and HRP conjugated anti-β-actin (1:10,000), (AC-15; A3854, Sigma Aldrich, Bangalore, India) The blots were washed with PBS-T and incubated with HRP conjugated anti-rabbit IgG (AP307P) secondary antibody (Sigma-Aldrich, Bangalore, India) for 1 h at room temperature. Post-incubation, the blots were washed thrice with PBS-T and developed using an ECL super signal west pico substrate kit (Pierce Biotechnology, Waltham, MA, USA) on X-ray films. The X-ray images were scanned by using a Canon image CLASS scanner (Model No. MF232w) and the expression levels of the probed proteins were calculated by using densitometry. The bands were analyzed and the intensity was quantified using Image J analysis software. β-actin was used as a loading control for immunoblotting and the band intensity values were normalized with β-actin.

### 2.4. RNA Isolation and Quantitative-Real Time PCR

To determine the IL-33 mediated changes in the mRNA expression of *IL-1α*, *IL-5*, *IL-13*, *IL-17A* and *NFκB*, THP-1 monocytes were stimulated for 2, 4, 6, 8 and 10 h, respectively. Total RNA was extracted using a RNeasy mini kit (Qiagen, Hilden, Germany) according to the manufacturer’s protocol. The cDNA was prepared from 200 ng of RNA from each timepoint using a PrimeScript cDNA synthesis kit (RR037A, Takara Bio, New Delhi, India) according to the manufacturer’s instructions. A q-PCR was performed on the cDNA using SYBR Green Master Mix (Takara Bio) as per the manufacturer’s recommendations. The gene-specific primers used in the study were purchased from (Sigma-Aldrich, St. Louis, MO, USA); details are provided in Supplementary Table S1. The cycle thresholds (Ct) of the target genes were normalized to Ct values of GAPDH, which was used as a reference gene; unstimulated cells were used as a control to calculate the fold change. The relative expression of the target gene was represented as fold change and calculated based on the 2^−ΔΔCT^ method [21]. The data are represented as mean ± SEM from three independent experiments with * *p* < 0.05 considered significant.

### 2.5. Sample Preparation for Mass Spectrometry

In-solution digestion of protein lysates were carried out as previously described [22]. Briefly, the protein lysate from each condition (~1 mg) was subjected to reduction and alkylation using 10 mM dithiothreitol (DTT) (I6125, Sigma Aldrich, Bangalore, India) and 20 mM iodoacetamide (IAA) (D9779, Sigma Aldrich, Bangalore, India) respectively. The protein lysates were subjected to enzymatic digestion with trypsin (1:20) (modified sequencing grade; Promega, Madison, WI, USA) for 16 h at 37 °C. After the confirmation of the tryptic digestion, the peptide samples were lyophilized, resuspended in 7 mM TEABC (pH 9) and labeled using a TMT 10 plex kit (Thermo Fisher Scientific, Rockford, IL, USA, 90110). The TMT-labeled samples from each condition were pooled and fractionated using basic pH reversed-phase liquid chromatography (bRPLC). The labeled peptides were loaded onto an XBridge BEH C18 Column (Waters Corporation, Milford, MA, USA) and resolved with an increasing gradient of 7 mM TEABC and 90% acetonitrile (pH 9) over a 30 min duration, at a flow rate of 1 mL/min, using an Agilent 1100 LC system. A total of 96 fractions were collected, which were finally concatenated to 12 fractions. In total, 10% of the pooled fractions were aliquoted for total proteome analysis and the remainder for phosphopeptides enrichment. All the fractions were evaporated to dryness, desalted using C18 cartridges (66883-U, Empore, Bellefonte, PA, USA), vacuum dried and stored in a −80 °C deep freezer until further analysis.

### 2.6. TiO_2_-Based Phosphopeptide Enrichment

The TiO_2_-based phosphopeptide enrichment of all three biological replicates (36 fractions) was carried out as described by Larsen et al. [23]. The TiO_2_ beads (Titansphere; GL Sciences, Inc., Tokyo, Japan) were suspended in DHB solution (80% ACN, 1% TFA, and 5% 2,5-Dihydroxybenzoic acid) at room temperature for 1 h. Each fraction was then resuspended in 5% DHB solution and incubated with TiO_2_ beads for 30 min at room temperature with gentle rotation. TiO_2_ beads enriched with phosphopeptides were washed thrice with DHB solution and then with 40% ACN twice. The peptide mixture was acidified using 1% Trifluoroacetic acid (TFA) and desalted using a C18 Sep-Pak cartridge (WAT051910, Waters Corporation, Milford, MA, USA). The peptides were concentrated by vacuum centrifugation and subjected to a C18 StageTip cleanup before mass spectrometry analysis.

### 2.7. LC-MS/MS Analysis

The total proteome and phosphopeptide-enriched fractions were reconstituted in 0.1% formic acid and analyzed on a Thermo Scientific Orbitrap Fusion Tribrid mass spectrometer (Thermo Fisher Scientific, Bremen, Germany), coupled to an Easy-nLC-1200 nanoflow liquid chromatography system (Thermo Scientific, Bremen, Germany). The reconstituted peptides were loaded onto a 2 cm trap column (nanoViper, 3 µm C18 Aq) (Thermo Fisher Scientific) and resolved on a 15 cm analytical column (nanoViper, 75 µm silica capillary, C18 Aq) at a flow rate of 300 nL/min. The solvent gradients were set as follows: a linear gradient of 5 to 30% solvent B (80% acetonitrile (ACN) in 0.1% formic acid) over 100 min followed by a stepped gradient of 60–100% solvent B for 4 and 7 min respectively. An MS analysis was performed on an Orbitrap mass analyzer in data-dependent acquisition (DDA) mode with a scan range of 400–1600 m/z mass range at 120,000 mass resolution at 200 m/z. The injection time was limited to 10 ms. For the MS2 analysis, the data were acquired at top speed mode with a duty cycle of 3 s and subjected to higher collision energy dissociation with a normalized collision energy of 32. The MS/MS scans were performed on an Orbitrap mass analyzer with a resolution of 30,000 at 200 m/z and a maximum injection time of 200 ms. Internal calibration was carried out using a lock mass option (m/z 445.1200025) from ambient air.

### 2.8. Identification of Peptides and Proteins

The Mass spectrometry data (.raw) were searched against the human RefSeq database (RefSeq94 containing 113,658) appended with 116 frequently observed contaminants using Mascot (v2.2) and SEQUEST HT search algorithms through the Proteome Discoverer platform (v2.2, Thermo Scientific, Bremen, Germany). The search parameters for both algorithms included a maximum of one missed cleavage, oxidation of methionine, phosphorylation at serine, threonine, tyrosine as dynamic modifications and carbamidomethylation at cysteine, TMT 6 plex Lysine and TMT 6 plex N-terminal as static modifications. A precursor mass error tolerance of 10 ppm and a fragment mass error tolerance of 0.05 Da were considered during the analysis. The data were searched against a decoy database and peptides identified at a <1% false discovery rate (FDR) were considered further for protein identification. The phosphorylation probability at each S/T/Y site was calculated using the PTM-RS node in the Proteome Discoverer; peptides with more than 75% site localization probability were considered for further analysis.

### 2.9. Bioinformatics Data Analysis

The high-confidence peptides identified using the Proteome Discoverer search results were further used for data normalization and quantitation. The TMT reporter ion abundances for all the proteins across multiple replicates were log-transformed and analyzed in limma v3.44.3 [24] using R (v4.0.2, https://www.r-project.org/, accessed on 26 December 2021). Using limma, the removal of the batch effect was carried out and the fold changes were computed. The treatment conditions were used as blocking factors in the linear model. Phosphopeptides with a log2-fold change ≥±0.58496 were considered to be differentially regulated. An online post-translational modification-profiling (PTM-Pro) (Version 2.0) tool (http://ptm-pro.inhouseprotocols.com/, accessed on 26 December 2021) was employed for summarizing the high-confidence PTMs with a ptmRS site probability >75%, in accordance with previous research [25].

Hypergeometric enrichment-based pathway analyses were carried out as described previously [26]. Briefly, R (R studio v. 1.3.1073, R v. 4.0.2, Bioconductor v. 3.11.1) scripts using clusterProfiler (v. 3.16.1) [27] and Reactome pathways [28] using the Reactome package (v. 1.32.0) [29] were used for the analyses. The pathway enrichment parameters included ‘human’ as the organism, 0.005 as the *p*-value cut-off, a Benjamini–Hochberg correction base *p*-value adjustment, a minimum gene set size of 10 and a q-value cut-off of 0.2. The pathway analysis results were plotted as dot plots in R using the ggplot2 package (v. 3.3.4). A gene ontology analysis was carried out using Enrichr (https://maayanlab.cloud/Enrichr/, accessed on 26 December 2021). A comparison with the kinase and phosphatase lists was carried out as previously described [30].

### 2.10. Bioinformatics and Network Analysis to Identify Regulated Kinases and Substrates

The list of identified phosphopeptides from the phosphoproteomics approach results was compared with existing research using kinome map enrichment (http://www.kinhub.org/kinmap/index.html, accessed on 26 December 2021). The list of identified kinases was searched and relevant kinases were highlighted on the kinome map. The kinase enrichment analyses were performed using the online tool, eXpression2Kinases (X2K; http://amp.pharm.mssm.edu/X2K/, accessed on 26 December 2021), for phosphoproteins enriched with genes known to interact with kinases [31].

### 2.11. Statistical Analysis

The data were expressed as mean ± standard deviation. The statistical significance was analyzed using the student’s *t*-test and one-way variance (ANOVA) analysis for multiple groups. The data were analyzed using GraphPad Prism (GraphPad Software Inc., San Diego, CA, USA) (version 5.0).

### 2.12. Data Availability

The mass spectrometry proteomics data were deposited in the ProteomeXchange Consortium [32] (http://proteomecentral.proteomexchange.org, accessed on 26 December 2021) via the PRIDE partner repository with the dataset identifier total proteome PXD024385, phosphoproteome PXD028673.

## 3. Results

### 3.1. Signaling Responses Mediated by IL-33 in Human Monocytes

**Figure 1:**
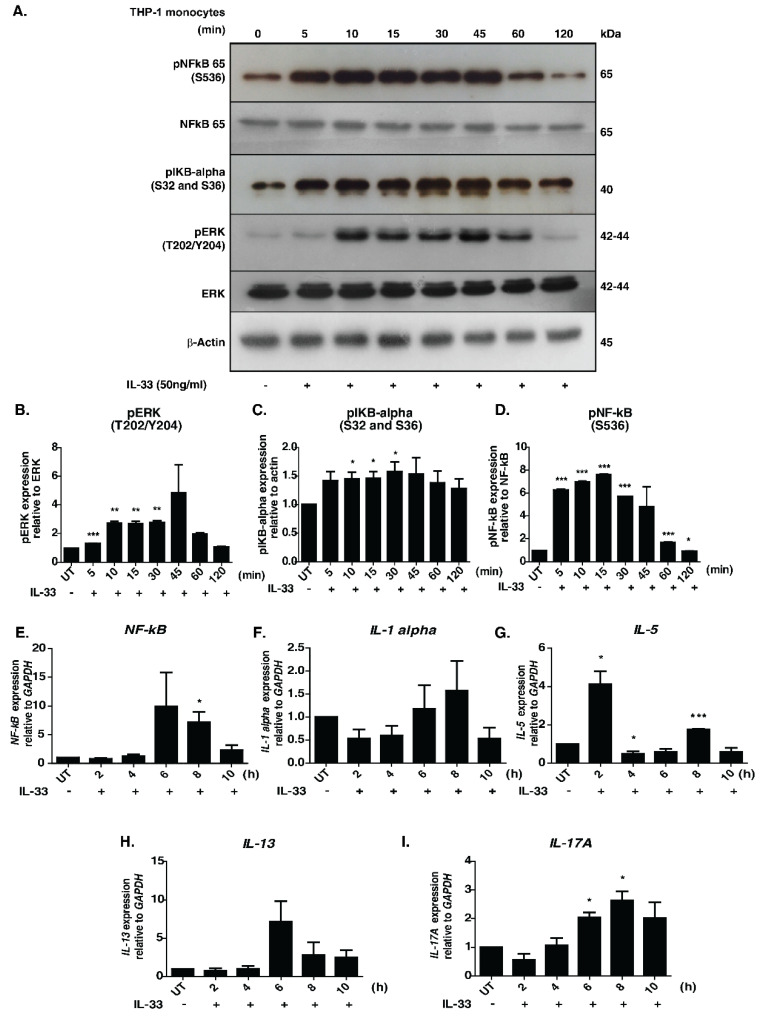
Effect of IL-33 treatment on the MAPK and NFκB signaling cascades in THP-1 monocytes. (**A**). Western blot analysis showing the effect of IL-33 (50 ng/mL) on the phosphorylation status of NF-kB-p65 (S536), IKBα (S32/36), total NFKB p65, total ERK1/2 and phosphorylated ERK1/2 (T202/Y204) at different time points (5, 10, 15, 30, 45, 60, and 120 min). Densitometry analysis of the Western blots for (**B**) ERK1/2, phosphorylated ERK 1/2, (**C**) phosphorylated IκBα, (**D**) NFκB-p65 and phosphorylated NFκB-p65. The relative fold changes are shown. * *p* < 0.05 compared to control (Mean ± SEM, *n* = 3). Further, IL-33 treatment induced mRNA expression of various cytokines, including (**E**) *NF-kB*, (**F**) *IL-1alpha*, (**G**) *IL-5*, (**H**) *IL-13* and (**I**) *IL-17A*. Quantitative real-time PCR was carried out after stimulating THP-1 monocytes with IL-33(50 ng/mL for varying durations (2, 4, 6, 8 and 10 h). The results are shown as fold change with respect to control cells (0 min). All the experiments were repeated in triplicates. * *p* < 0.05 compared to control (Mean ± SEM, *n* = 3). ** and *** denote medium and highly significant values respectively.

The signaling responses mediated by IL-33 were primarily studied in macrophages. To decipher the temporal influence of IL-33 in monocytes, we examined the phosphorylation dynamics of well-known downstream effectors, namely pERK, p65 and p IκBα, across seven-time points upon IL-33 stimulation. Increased phosphorylation of ERK1/2, IκBα and NFκB-p65 was observed in a time-dependent manner (Figure 1A–D). 

An increase in the phosphorylation status was observed as early as 10 min, with peak phosphorylation at 45 min post-stimulation. Our results, indicating activation of MAPK and NFκB modules, are therefore in agreement with those of previous research. The activation of NFκB signaling module in THP-1 cells by chemokines through a Gα (14/16)-mediated mechanism was reported previously [33]. It is possible that the IL-33-mediated NFκB signaling activation seen in our results is mediated by similar mechanisms. Further, we inhibited cells with U0126, a selective inhibitor of MAP kinase kinases-MEK1 and MEK2, to confirm the utility of the cell line model for downstream phosphoproteomics analysis. Our analysis revealed that stimulation by IL-33 followed by treatment with U0126 abolished phosphorylation of ERK1/2 but exerted minimal/no effect on the phosphorylation status of pIKBα and NFκB p65 (Supplementary Figure S1).

We further assessed the expression profile of the pro-inflammatory cytokines- *IL-1α*, *IL-5*, *IL-13*, *IL-17A*, and *NFκB*, which are known to be regulated upon IL-33 stimulation [7]. We observed an increased level of expression in all the transcripts except *IL-5* 6 h post-stimulation, with the levels of *IL-13* and *NF-κB* reducing gradually at 8 h post-stimulation. By contrast, the levels of *IL-1A* and *IL-17A* peaked at 8 h post-stimulation. Interestingly, *IL-5* mRNA showed a biphasic induction with increased expression observed 2 h post-stimulation and returning to basal levels by 4 h. Eight hours post-stimulation, the levels further increased, albeit to a lesser extent (Figure 1E–I). Our results demonstrate that IL-33 induces the expression of Th2-associated cytokines in monocytes. Overall, our results confirm the monocyte model’s utility for further downstream proteomic analysis.

### 3.2. Quantitative Temporal Analysis of Protein Phosphorylation Dynamics Upon IL-33 Stimulation

**Figure 2:**
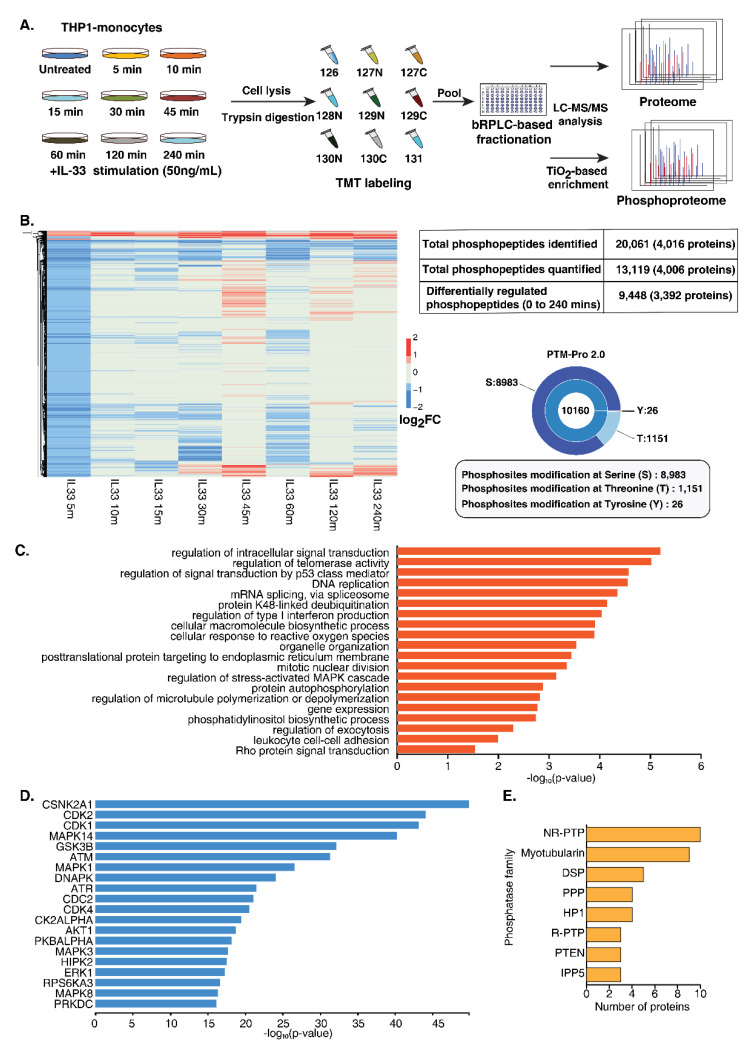
Temporal phosphoproteomics and proteomic analysis of THP-1 monocytes stimulated with IL-33. (**A**) Brief workflow depicting quantitative proteomic analysis of IL-33 stimulated THP-1 monocytes. THP-1 cells were stimulated with recombinant human IL-33 for indicated time points. Proteins were extracted from the cell lysates and subjected to in-solution trypsin digestion, followed by TMT-based chemical tagging for quantitative proteome profiling. The samples were analyzed on a Thermo Scientific™ Orbitrap Fusion™ Tribrid™ Mass Spectrometer. Mascot and SEQUEST algorithms were used for database searches. (**B**) Summary of the IL-33-induced phosphoproteome and total proteome. (**C**) Gene ontology analysis depicting the enriched biological processes for the IL-33-induced hyperphosphorylation events (**D**) Kinase enrichment analysis identified predicted upstream kinases for the phosphoproteins activated by IL-33. (**E**) Graph representing the top eight phosphatase families that were identified as being regulated by IL-33.

A multiplexed quantitative phosphoproteome analysis was performed to gain a comprehensive overview of protein dynamics and dissect the signaling responses mediated by IL-33. THP-1 monocytes were treated with rIL-33 at eight different time points (5, 10, 15, 30, 45, 60, 120 and 240 min). All the treatments were performed in triplicate. After treatment, the cells were harvested in an SDS lysis buffer, followed by trypsin digestion and a C18-based cleanup. The peptide digests from each timepoint were isobarically labeled using a 10 plex tandem mass tagging (TMT) labeling approach followed by the enrichment of phosphopeptides using TiO_2_-affinity enrichment. The enriched phosphopeptides were analyzed using an Orbitrap Fusion mass spectrometer (Figure 2A), resulting in the identification of 20,061 phosphopeptides mapping to 4016 proteins. PTM-pro tool was used to identify high-confidence PTM sites based on ptmRS probability cut-off (100%). Overall, we identified 8983, 1151 and 26 phosphorylated serine, threonine and tyrosine sites, respectively. The distribution of these PTMs sites is represented in Figure 2B. A total of 13,119 phosphopeptides mapping to 4006 proteins was quantified (Supplementary Table S2). Of these, 9448 unique phosphopeptides corresponding to 3392 proteins were regulated by IL-33 at least at one-time point (Supplementary Figure S2A).

To assess whether the proteome expression exerted any effect on the changes in the phosphorylation dynamics, we carried out global proteome profiling of IL-33-treated THP-1 monocytes at similar time points. We identified 8109 proteins, of which 3163 proteins overlapped with the phosphoproteins identified in the phosphoproteome data (Supplementary Table S3). Overall, minimal protein expression-level changes were observed across the time points.

A gradual increase in the extent of the phosphorylation of a number of phosphosites was observed upon IL-33 treatment from 5 min to 45 min. Notably, a decrease in phosphorylation was observed at 60 min for several of these proteins, followed by an increase at 120 min, likely indicating the biphasic activation of the signaling response. Overall, 55 phosphopeptides corresponding to 50 proteins showed sustained hyperphosphorylation, while 59 phosphopeptides corresponding to 59 proteins showed sustained hypophosphorylation across all the time points. ARAF (S257), a Ser/Thr kinase involved in RAS-MAPK cascade, was consistently hyperphosphorylated upon IL-33 stimulation. We also observed several phosphorylation sites whose phosphorylation levels were downregulated across timepoints, especially with an increased number observed at 5 min followed by 60 min (Supplementary Figure S2A). This suggests that IL-33 likely regulates the activity of protein phosphatases that plausibly serve as a feedback system to balance the signaling response mediated by the activation of the IL-33/ST2 axis. Furthermore, several proteins phosphorylated at multiple sites demonstrated different kinetics. For example, INPP5D, a phosphatase previously shown to be regulated by IL-33 was found to be quantified at three sites; phosphorylation at T963 and S971 peaked at 45 min, whereas S243 peaked at 120 min and S1039 peaked at 240 min post-stimulation, strongly suggesting the activity of multiple regulators on a single protein.

The gene ontology-based enrichment analysis of upregulated and downregulated phosphosites revealed significant enrichment of biological processes, including DNA repair, response to reactive oxygen species, cell cycle checkpoints and mRNA splicing (Figure 2C and Figure S2B). A subset of phosphoproteins, including transcription factors and protein kinases known to play a role in DNA damage response, cell differentiation and inflammatory immune response proteins is provided in Table 1.

### 3.3. Dynamic Regulation of Protein Kinases and Phosphatases

Among the IL-33-regulated phosphoproteins, 171 protein kinases and 60 phosphatases were identified whose phosphorylation status was significantly regulated across timepoints. Furthermore, multiple phosphorylation sites on several protein kinase family members were observed. These included AGC (21), CAMK (21), CMGC (22), STE (30), TK (17), TKL (10), Atypical Alpha-type (3), PI3/PI4-kinase (6), RIO-type (3), CK1 (4), NEK (4), and 29 belonging to other protein kinase families (Supplementary Figure S2C). This indicates the involvement of signaling cascades, including the known MAPK signaling cascade RAF1 (S43/S301), MAPK1 (T185/T187), IKK beta (S672/S697), PI3K/AKT module, calcium-regulated signaling and intracellular signaling mediated by cyclic nucleotides, phospholipids and calcium (AGC). Interestingly, the NEK Ser/Thr protein kinase family members known to play a vital role in cell cycle regulation and DNA damage response were differentially regulated [34,35]. Notably, NEK9 was hyperphosphorylated at S29 at all time points except at 5 min. We also observed differential phosphorylation at multiple serine and threonine residues on tyrosine kinases.

An upstream kinase analysis of the differentially regulated phosphoproteins using X2Kweb, which ranks enriched kinases based on known kinase–substrate interactions [36] further revealed enrichment of the kinases involved in MAPK signaling (MAPK1, MAPK3, MAPK8 (JNK) MAPK14 (p38)) and PI3K/AKT module (GSK3B, AKT1, and RPS6KA3) thereby confirming the involvement of these IL-33 regulated cellular signaling networks in human monocytes. Furthermore, we observed a significant enrichment of CSNK2A1, members of cyclin-dependent kinases (CDK1, CDK2 and CDK4) and kinases involved in DNA damage response (ATM, ATR and DNAPK) (Figure 2D).

Along with kinases, we also identified several phosphatases regulated by IL-33. Among the 60 phosphatases that were found to be regulated by IL-33 across various time points, 41 belonged to 8 phosphatase families, including non-receptor protein tyrosine phosphatase (NR-PTP) (10), Myotubularin (9), DSP (5), PPP (4), HP1 (4), receptor tyrosine phosphatase (R-PTP) (3), PTEN (3) and IPP5 (3) (Figure 2E). The rest were distributed across 14 phosphatase families. Interestingly, several phosphatases belonging to the NR-PTP and myotubularin families were found to be regulated by IL-33. These included the sustained hyperphosphorylation of PTPN14 (S620) 30 min post-stimulation, hypophosphorylated levels of PTPN2 (S304) and the hyperphosphorylation of MTMR3 (T731) and MTMR10 (S607) 45 min post-stimulation, among others. NR-PTPs have been demonstrated to play distinct roles in immune cell regulation [37,38]. The myotubularin-related protein family is a large family of phosphatases primarily involved in vacuolar transport and membrane trafficking functions [39]. They have been known to interact with several proteins, including transcriptional regulators, that are involved in the regulation of DNA repair, cell death and growth [40].

Further, a comparison with human transcription factors (TF) with known motifs [41] revealed 378 phosphopeptides corresponding to 178 TF to be regulated by IL-33. The majority of these demonstrated increases in phosphorylation 30 min post-stimulation, with peak phosphorylation observed at 45 min post-stimulation. In addition to identifying TFs known to be regulated by IL-33, such as NFKB, ATF2, FOS, JUND, NFATC1 GATA2 and FOXO1, our analysis identified the hyperphosphorylation of novel TFs, such as DNMT1 (S154) and members of the forkhead box protein family, such as FOXO1 (S256, S287), FOXK1 (S441, 445), FOXM1 (S522), FOXP2 (S591), among others. FOXO1 is a critical cell death regulator and acts downstream of CDK1, PKB/AKT1 and STK4/MST1 kinases [42,43]. We observed increased phosphorylation at S256, which has been demonstrated to be essential for its nuclear export and, therefore, the inhibition of its transcription factor activity [44]. We identified the hyperphosphorylation of several sites on Zinc finger E-box binding homeobox 1 protein (ZEB1), which has recently been demonstrated to be a vital regulator of DNA damage response and EMT-mediated cell plasticity [45]. ZEB1 regulates DDR by forming a complex with p300/PCAF and is a direct target of ATM kinase, thereby promoting its stability. A recent study implicated the role of GATA transcription factors, specifically GATA2 downstream of IL-33 signaling, in regulating the development of iron-recycling macrophages [46]. We observed decreased phosphorylation at S192 at early time points. However, the phosphorylation reached basal levels at 45–60 min and then reduced, indicating transient activation. Phosphorylation at S192 is p38/ERK-dependent and is required for GATA2-mediated transcriptional activation [47]. A similar trend was also observed for POU2F1 (S448). In conclusion, we found that IL-33 regulated several phosphorylation sites on transcription factors. However, the precise role of these transcription factors in IL-33 signaling needs to be determined.

### 3.4. Delineating IL-33 Signaling and Signaling Modules in Monocytes

**Figure 3:**
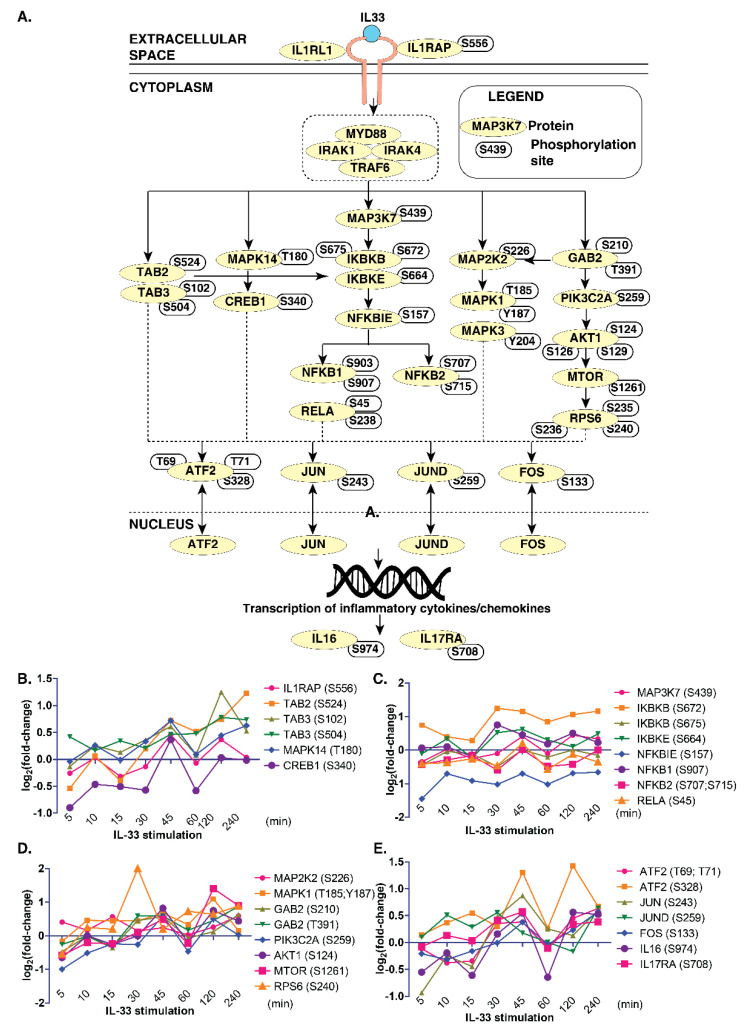
A snapshot of IL-33 signaling in THP-1 monocytes. (**A**) Brief schematic of the IL-33 pathway showing proteins and phosphosites identified by the phosphoproteome analysis. Temporal changes in IL-33 pathway phosphopeptides of: (**B**) TAB2/3 and MAPK14 modules; (**C**) IKBK-NFKB1 modules; (**D**) MAPK and PI3K/AKT modules; and (**E**) transcription factors and interleukins.

Next, we compared the phosphoproteomics data from this study with the previously published map of the IL-33 signaling pathway [7] to determine the temporal signaling responses of IL-33-specific signaling modules in monocytes. We observed an overlap between several critical signaling modules, including the ERK1/2 (MAPK1/3) module, PI3K-AKT, NFκB and p38MAPK (MAPK14) modules in THP-1 monocytes (Figure 3A).

However, we could not identify phosphosites pertaining to the JAK-STAT pathway in our data. Further, we looked closely at the temporal phosphorylation levels of proteins belonging to these signaling modules (Figure 3B–E). The ERK1/2 signaling module consisted of MAPK1(T185, Y187) and MAPK3 (Y204), as well as their kinase, MAP2K2 (S226). The phosphoproteins in the PI3K-AKT signaling arm consisted of GAB2 (S210, T391), PIK3C2A (S259), AKT1 (S124, S126, S129), MTOR(S1261) and RPS6 (S235, S236 and S240). The NFκB signaling arm consisted of IKBKB (S672), IKBKE (S664), NFKBIE (S157), NFKB1 (S903, S907), NFKB2 (S707, S715) and RELA (S45, S238). The p38 arm consisted of MAPK14 (T180) and CREB1 (S340). Overall, our data indicate the existence and functional activation of IL-33-regulated signaling modules that were also previously identified in the context of macrophages in monocytes.

### 3.5. IL-33 Signaling Impacts DNA Damage/Repair Pathways in Monocytes

**Figure 4:**
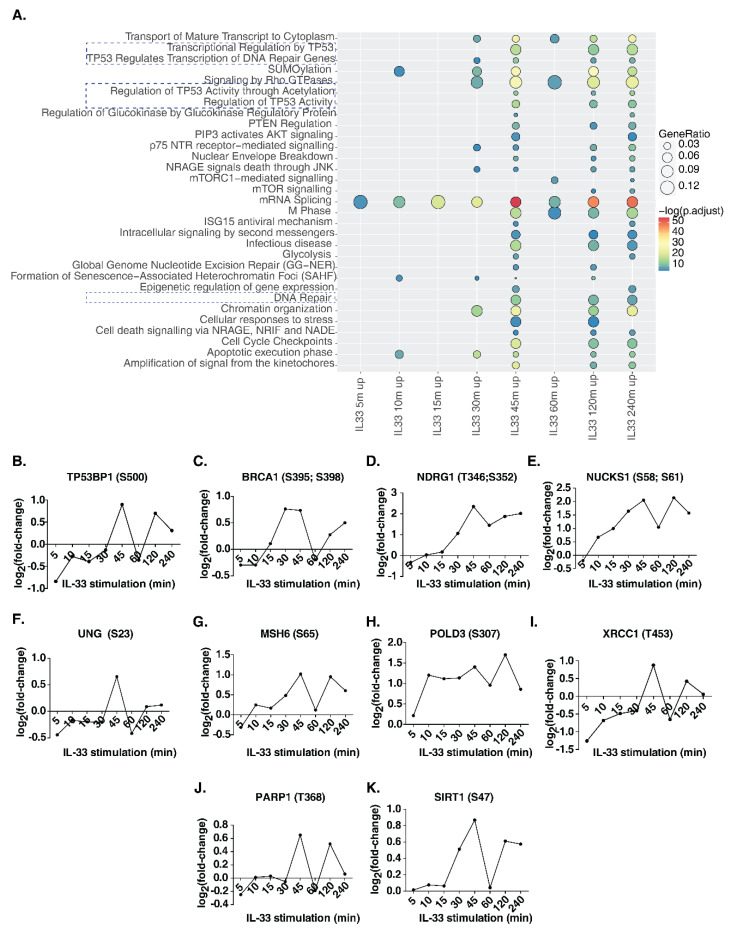
(**A**) Reactome pathway analysis of upregulated phosphoproteins showed induction of several signaling pathways including DNA damage and DNA repair pathways (highlighted with dotted lines). Further detailed analysis identified specific phosphosite changes in several DNA damage/repair proteins including (**B**) TP53BP1, (**C**) BRCA1, (**D**) NDRG1, (**E**) NUCKS1, (**F**) UNG, (**G**) MSH6, (**H**) POLD3, (**I**) XRCC1, (**J**) PARP1 and (**K**) SIRT1.

In addition to classical IL-33 signaling phosphorylation events, we observed several proteins involved in key cellular processes that were also regulated by IL-33. To identify signaling pathways impacted by IL-33 in THP-1 monocytes, we carried out a signaling pathway analysis for the hyperphosphorylated proteins identified at each time point between 5 and 240 min (Figure 4A, Supplementary Table S4). Between 5 and 30 min post-stimulation, relatively few pathways, such as mRNA splicing, SUMOylation and apoptosis, were observed to be enriched. Interestingly, from 30 min post-stimulation, Rho GTPase signaling, chromatin organization, NRAGE signaling and p75 NTR signaling were found to be significantly enriched. Further, the enrichment of DNA damage/repair pathways occurred in the intermediate phase between 30 and 45 min and continued until 240 min post-stimulation. These included transcriptional regulation by TP53, regulation of TP53 activity and DNA repair pathways. We then analyzed the temporal regulation of phosphorylation on DNA damage/repair pathway proteins, including tumor protein p53 binding protein 1(TP53BP1), BRCA1 DNA repair-associated (BRCA1), N-myc downstream regulated 1 (NDRG1), nuclear casein kinase and cyclin-dependent kinase substrate 1 (NUCKS1), uracil DNA glycosylase (UNG), mutS homolog 6 (MSH6), DNA polymerase delta 3, accessory subunit (POLD3), X-ray repair cross-complementing 1 (XRCC1), poly(ADP-ribose) polymerase 1 (PARP1) and sirtuin 1 (SIRT1) (Figure 4B–K). Our analysis indicates that several phosphosites on proteins known to be involved in DNA damage/repair pathway show a biphasic mode of phosphorylation, with a substantial increase observed at 45 min post-IL33-stimulation, dipping at 60 min, and increasing again at 120 min, indicating a feedback regulation of this response. In addition, we checked whether there were any changes in the phosphosites on the proteins belonging to the DNA damage sensing and activation pathway in response to IL-33 (Supplementary Figure S4). The primary sensors of DNA damage, including Nibrin (NBN or NBS1), MRE11 homolog, double-strand break repair nuclease (MRE11), and RAD50 double-strand break repair protein (RAD50), showed hyperphosphorylation between 30 and 45 min after IL-33 stimulation. In addition, DEAD-box helicase 41 (DDX41), interferon-gamma inducible protein 16 (IFI16), caspase recruitment domain family member 9 (CARD9), ATM serine/threonine kinase (ATM) and interferon regulatory factor 3 (IRF3) showed increased levels of phosphorylation at a few sites at 45 min while ATR serine/threonine kinase (ATR), X-ray repair cross-complementing 5 (XRCC5 or Ku80) and X-ray repair cross-complementing 6 (XRCC6 or Ku70) showed decreased phosphorylation levels. Overall, these data indicate the induction of DNA damage response between 30 and 45 min post-IL-33 stimulation. To confirm the findings, we compared our results with the dataset on the IL-33 phosphoproteomic landscape of murine macrophages published previously by our group [15]. Several DNA damage response proteins, including Ndrg1 (S389), Rad17 (S70), Msh6 (S77), Nbn (S398), Card9 (S425) and Ddx41 (S66, S68, T59), among others, showed hyperphosphorylation, albeit at lower levels due to the low duration (10 min) of IL-33 stimulation.

## 4. Discussion

Signaling responses mediated by alarmins that are released upon cellular stress or tissue injury are vital to alert the immune system of impending danger and induce innate/inflammatory and adaptive immune responses [48,49]. IL-33, a pleiotropic nuclear-associated cytokine, is one such alarmin. It is constitutively expressed in epithelial barrier tissue and hematopoietic cells and rapidly released upon cellular damage [50]. Further, studies have demonstrated an increased expression of IL-33 in both primary and THP1 monocytes in response to stimulation with TLR4 and TLR2 agonists [51], indicating pathogen-associated release. In the extracellular milieu, IL-33 initiates innate/inflammatory and adaptive immune responses that are dependent on a number of signaling events. Although IL-33 is a potent regulator of IL-33/ST2 signaling in monocytes and macrophages, only a limited number of studies have explored time-dependent IL-33-mediated signaling dynamics in THP-1 monocytes. In the current study, we aimed to elucidate the signaling dynamics mediated by IL-33 in monocytes on a global scale. Our in-depth quantitative phosphoproteomics analysis revealed that early, intermediate and late IL-33 signaling is characterized by distinct signaling profiles.

The cytokine IL-33 signals primarily through the activation of signaling modules, including MAPK signaling cascades comprising ERK, JNK and p38 (MAPK14) modules, PI-3K/AKT/mTOR and NFκB pathway in various hematopoietic cell types. Our data showed that these signaling modules are also activated in monocytes. Further, IL-33 signaling culminates in the activation of transcription factors, including JUN, ATF2 and RELA (NFκB p65), resulting in the production of a range of immunomodulatory cytokines. The temporal stimulation of THP1 monocytes with IL-33 induced an increased expression of *IL-5* mRNA, a Th2 cytokine, as early as 2 h post-stimulation, whereas the expression of *IL-1α*, *IL-13* and *IL-17A* mRNA increased after between 6 and 8 h of stimulation. This is in line with previous findings that showed that group 2 innate lymphoid cells (ILC2s) and CD4+ T helper 2 cells secrete large quantities of IL-5 and IL-13 in response to IL-33 [13]. Interestingly, the expression of *IL-5* mRNA revealed a biphasic induction in monocytes. To our knowledge, this constitutes the first report of IL-33-induced cytokine expression dynamics in monocytes.

We further observed a robust induction and dynamic regulation of phosphorylation patterns across proteins by IL-33. Notably, these are involved in various cellular processes, including response to external stimuli, leukocyte adhesion, regulation of microtubule polymerization, Rho protein-mediated signal transduction as well as hitherto unknown processes. We have previously shown that IL-33 mediates the activation of cdc42/Rho signaling in murine macrophages [15], which is vital for immune cell motility, polarity and directed migration [52]. Although the extent of phosphorylation was not drastic, especially for the MAPK activation sites, the upstream kinase enrichment analysis revealed significant enrichment of MAPKs including ERK1/2, MAPK14 and MAPK8 (JNK1), strongly indicating the activation of these signaling cascades in monocytes, in addition to the other well-known signaling modules, including PI3K/AKT module and calcium-regulated signaling essential for monocytic immune function.

In addition to canonical IL-33 signaling modules, we focused on the influence of IL-33 on the kinome and phosphatome. Kinases and phosphatases constitute important cellular machinery that regulates phosphorylation and therefore signaling dynamics [30,53]. More importantly, these can serve as candidates for targeted therapy for diseases where aberrant IL-33 signaling is observed. Our data revealed that IL-33 regulated 171 protein kinases and 60 phosphatases in the early, intermediate and late phases of signaling. Apart from the well-known MAPK1 and PI3K/AKT, our data indicated that IL-33 regulated several NEK Ser/Thr protein kinase family members that play a crucial role in cell cycle regulation and DNA damage response. Among the differentially phosphorylated protein phosphatases, we observed an overrepresentation of phosphopeptides mapping to non-receptor protein tyrosine phosphatase subfamily (or PTPNs). Several PTPNs have been previously implicated in cytokine signaling [54]. For example, PTPN2 was found to regulate IFNgamma-induced cytokine signaling in THP1 monocytes through decreased STAT1 and STAT3 activity and the secretion of IL-6 and MCP-1 [55]. Further, splenic macrophages from Ptpn2-null mice were found to be hyperresponsive to LPS, suggesting that Ptpn2 is a negative regulator of inflammation [56]. These suggest that PTPN2 could play a role in inflammatory processes in monocytes. In addition, we observed altered phosphorylation on PTPN14, which has recently been demonstrated to initiate cytokine storm and aggravate acute liver failure by interacting with and targeting SOCS7, a negative regulator of NF-κB signaling pathway to proteasomal degradation [57]. Whether IL-33 mediated alterations in the phosphorylation status of these PTPNs, as well as other phosphatases identified in this study, play a role in immune modulation remains to be determined.

Transcription factors are well-known regulators of gene expression. Several of these play important roles in monocyte biology regulating development [58,59], regulating monocyte-to-macrophage differentiation [60,61] and causing the overexpression of cell-specific cytokines and chemokines [62]. In our study, IL-33 was found to regulate the phosphorylation dynamics of 178 transcription factors, including several members of the forkhead-box (FOX) family of transcription factors, which play a critical role in immunoregulation in several immune cell lineages. Furthermore, RNA-binding proteins such as YBX-1, which plays a vital role as a key mediator of immune regulators involved in bacterial and sterile inflammation [63], were also found to be differentially regulated. The regulation of several transcription factors across various signaling phases in our study suggests widespread transcriptional control by IL-33.

Finally, our data showed that IL-33 impacted several important processes and signaling pathways that, we speculate, could explain its role as an alarmin in monocytes. Processes and pathways pertaining to DNA damage response, DNA repair, response to reactive oxygen species, cell cycle checkpoints and mRNA splicing were found to be regulated by IL-33. Unlike IL-33 signaling in macrophages, in monocytes it appears that there are relatively few significantly enriched processes and pathways at the early phase in comparison with mid and late signaling phases, suggesting possible feedback mechanisms. Importantly, we identified several novel downstream effectors differentially phosphorylated at multiple sites, including NOC2L (S49, S56), NCOA2 (S736), USP16 (S415), NUCKS1 (S19, S181), NCOR2 (S149, S152), TP53BP1 (S366), SMARCA2 (S1377), MTDH (S308), THRAP3 (S248), YAP1 (T110), EIF4G1 (S1147), BCLAF1 (S531) and ACTL6A (S233), that play major role in DNA damage response, cell differentiation and inflammatory immune response.

IL-33 signaling has been previously shown to recruit monocytes to the lung interstitium through the upregulation of chemokines, including CCL2, CCL7 and CCL22 [64]. IL-33 has also been known to regulate gene expression in different ways. Intracellular IL-33 can regulate gene expression by binding to the nucleosome and modulating chromatin [65,66,67]. Further, it can act as a transcription factor by binding to transcriptional repressors, such as SUV39H1 histone lysine methyltransferase [65], leading to the repression of IL1R4 and IL6 [68]. In addition, IL-33 can bind to transcription factors, such as NFκB, to regulate incoming proinflammatory signals [69]. IL-33 has also been shown to regulate RELA (NFκB-p65) through binding [70]. In conclusion, we speculate that IL-33 plays a role as an alarmin in monocytes by inducing monocyte recruitment, activating DNA damage sensing and repair mechanisms and regulating gene expression. Several studies have indicated reciprocal interactions between DNA repair/damage responses and a few aspects of immunity, with signaling crosstalk between them, allowing injured cells to repair damaged DNA or communicate their damage to the microenvironment [71]. In this study, we observed that IL-33 regulated proteins belonging to both DNA damage response and DNA repair pathways as well as DNA damage sensing and activation pathways through phosphorylation. 

This study provides a detailed understanding of different IL-33 induced signaling mechanisms that could serve as potentially useful resources with which to study IL-33 function in the context of inflammatory and pathological conditions. These discoveries can further serve as a baseline resource for the development of therapeutic targets for diseases in which IL-33 signaling plays a role, including bacterial and viral diseases and cancers.

## Figures and Tables

**Table 1:** Partial list of the differentially regulated phosphoproteins upon IL-33 stimulation.

Gene Symbol		Log2(Fold Change) (IL-33-Treated/Untreated)							
	Phosphosite Modification	5 min	10 min	15 min	30 min	45 min	60 min	120 min	240 min
NOC2L	(S49); (S56)	0.94	1.47	1.02	1.61	1.92	1.39	2.28	1.17
NCOA2	(S736)	1.11	1.89	1.30	1.80	1.97	1.68	1.75	1.57
USP16	(S415)	0.67	1.04	0.72	1.32	1.91	0.67	1.75	0.76
NUCKS1	(S19)	0.92	1.93	1.40	2.27	1.83	1.76	1.73	1.47
NCOR2	(S149); (S152)	0.85	1.15	0.88	1.02	1.88	0.79	1.62	1.10
TP53BP1	(S366)	0.71	0.74	0.77	0.96	1.60	1.33	1.38	1.59
SMARCA2	(S1377)	0.58	1.20	0.87	1.46	1.13	1.00	1.31	1.03
MTDH	(S308)	0.73	0.95	0.50	1.16	1.58	0.83	1.43	0.52
THRAP3	(S248)	0.77	1.06	0.73	0.96	1.23	0.70	1.22	0.82
YAP1	(T110)	0.77	1.22	1.49	0.90	0.70	1.29	1.16	0.74
EIF4G1	(S1147)	0.97	1.47	1.18	1.72	1.85	1.02	1.73	1.21
BCLAF1	(S531)	0.74	1.14	0.96	1.16	1.22	0.89	1.44	1.14
ACTL6A	(S233)	0.62	1.16	0.75	1.10	0.92	0.80	1.07	0.82

## Data Availability

The mass spectrometry proteomics data were deposited in the Proteo-meXchange Consortium (http://proteomecentral.proteomexchange.org, accessed on 26 December 2021) via the PRIDE partner repository with the dataset identifier total proteome PXD024385, phosphoproteome PXD028673.

## Supplementary Materials

The following are available online at https://www.mdpi.com/article/10.3390/cells11010138/s1. Supplementary Table S1: List of forward and reverse primers used for RT-PCR. Supplementary Table S2: List of phosphoproteins and peptides identified from THP-1 monocytes stimulated with IL-33 for 5, 10, 15, 30, 45, 60, 120 and 240 min. Supplementary Table S3: List of proteins identified from THP-1 monocytes stimulated with IL-33 for 5, 10, 15, 30, 45, 60, 120 and 240 min. Supplementary Table S4: Complete list of Reactome signaling pathways enriched for phosphoproteins upregulated in response to IL-33. Supplementary Figure S1: (A) Western blot analysis showing the effect of IL-33 (50 ng/mL) and U0126 inhibition on the phosphorylation of NF-kB-p65, phosphorylated NF-kB-p65(S536), phosphorylated IKBα (S32/36), total ERK1/2, phosphorylated ERK1/2 (T202/Y204) between 2-240 min. For densitometry analysis of the Western blots, see (B) for ERK1/2 and phosphorylated ERK 1/2, (C) for phosphorylated IκBα and (D) for NFκB-p65 and phosphorylated NFκB-p65. The relative fold changes are shown. * *p* < 0.05 compared to control (mean ± SEM, *n* = 3). Supplementary Figure S2: (A) Summary of IL-33-induced hyper and hypophosphorylated phosphopeptides. (B) Gene ontology analysis depicting the enriched biological processes for the IL-33-induced hypophosphorylation events. (C) Kinase tree depicting identification of kinases in THP-1 cells upon IL-33 mediated signaling. Supplementary Figure S3: Kinase-substrate networks induced by IL-33. eXpression2Kinases analysis was carried out for hyper-phosphorylated proteins induced by IL-33 in THP-1 monocytes. (A) Protein-transcription factor networks induced by IL-33. (B) Network of kinases and transcriptional factors enriched in hyper-phosphorylated data set in IL-33-stimulated THP-1 monocytes. Supplementary Figure S4: Temporal changes in phosphosites on proteins belonging to the DNA damage sensing and activation pathway in response to IL-33. (A) NBN (NBS1), (B) MRE11, (C) RAD50, (D) pDDX41, (E) ATM, (F) ATR, (G) IFI16, (H) XRCC5 (Ku80), (I) XRCC6 (Ku70), (J) CARD9 and (K) IRF3
