# Supplementary material for: Temporal Quantitative Phosphoproteomics Profiling of Interleukin-33 Signaling Network Reveals Unique Modulators of Monocyte Activation
Source: Cells. 2022 Jan 1;11(1):138. doi: 10.3390/cells11010138 (PMC8749991; doi:10.3390/cells11010138)

Supplementary Data

Supplementary Tables

Supplementary Table S1. List of forward and reverse primers used for RT-PCR

Supplementary Table S2. List of phosphoproteins and peptides identified from THP-1 monocytes stimulated with IL-33 for 5, 10, 15, 30, 45, 60, 120, and 240 minutes.

Supplementary Table S3. List of proteins identified from THP-1 monocytes stimulated with IL-33 for 5, 10, 15, 30, 45, 60, 120, and 240 minutes.

Supplementary Table S4. Complete list of Reactome signaling pathways enriched for phosphoproteins upregulated in response to IL-33

Supplementary Figures

Supplementary Figure S1. A. Western blot analysis showing the effect of IL-33 (50 ng/ml) and U0126 inhibition on the phosphorylation of NF-kB-p65, phosphorylated NF-kB-p65(S536), phosphorylated IKBα (S32/36), total ERK1/2, phosphorylated ERK1/2 (T202/Y204) between 2-240 minutes Densitometry analysis of the Western blots for **B.** ERK1/2, phosphorylated ERK 1/2, C**.** phosphorylated IκBα, **D.** NFκB-p65 and phosphorylated NFκB-p65. The relative fold changes are shown. *P < 0.05 compared to control (Mean ± SEM, n = 3).

Supplementary Figure S2. A. Summary of IL-33-induced hyper and hypophosphorylated phosphopeptides **B.** Gene ontology analysis depicting the enriched biological processes for the IL-33-induced hypophosphorylation events. **C.** Kinase tree depicting identification of kinases in THP-1 cells upon IL-33 mediated signaling.

Supplementary Figure S3. Kinase-substrate networks induced by IL-33. eXpression2Kinases analysis was carried out for hyper-phosphorylated proteins induced by IL-33 in THP-1 monocytes. **A.** Protein-transcription factor networks induced by IL-33. **B.** Network of kinases, and transcriptional factors enriched in hyper-phosphorylated data set in IL-33-stimulated THP-1 monocytes.

Supplementary Figure S4. Temporal changes in phosphosites on proteins belonging to the DNA damage sensing and activation pathway in response to IL-33. A. NBN (NBS1), B. MRE11, C. RAD50, D. pDDX41, E. ATM, F. ATR, G. IFI16, H. XRCC5 (Ku80), I. XRCC6 (Ku70), J. CARD9, and K. IRF3

Supplementary Figure S1

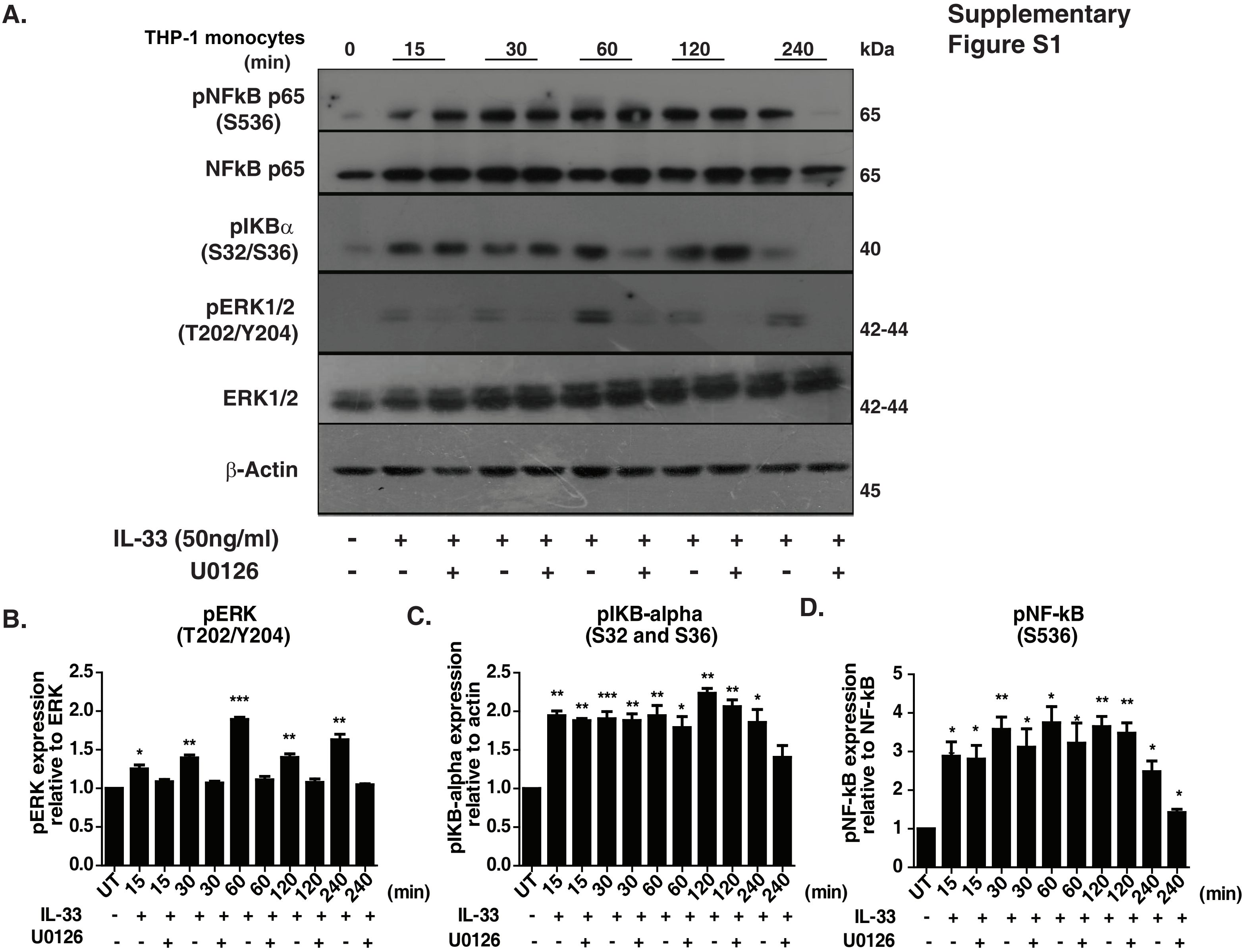

Supplementary Figure S2

A.

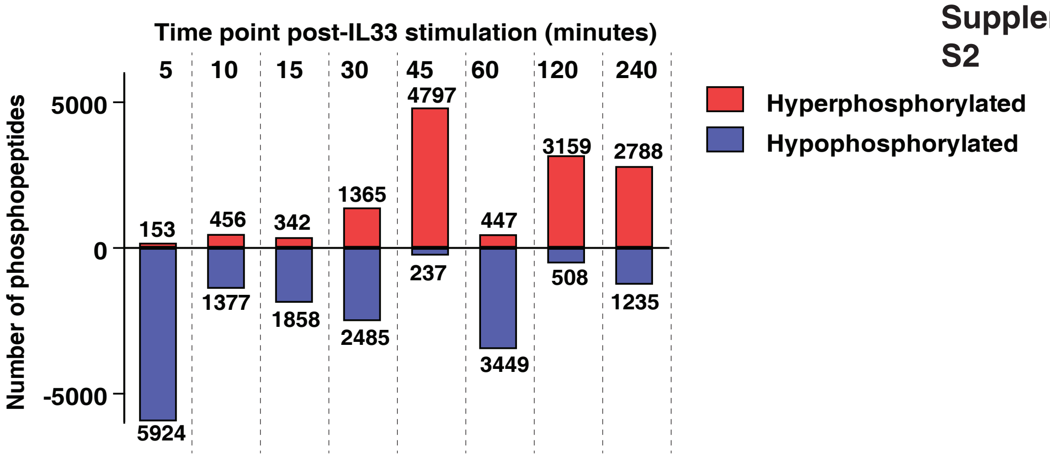

B.

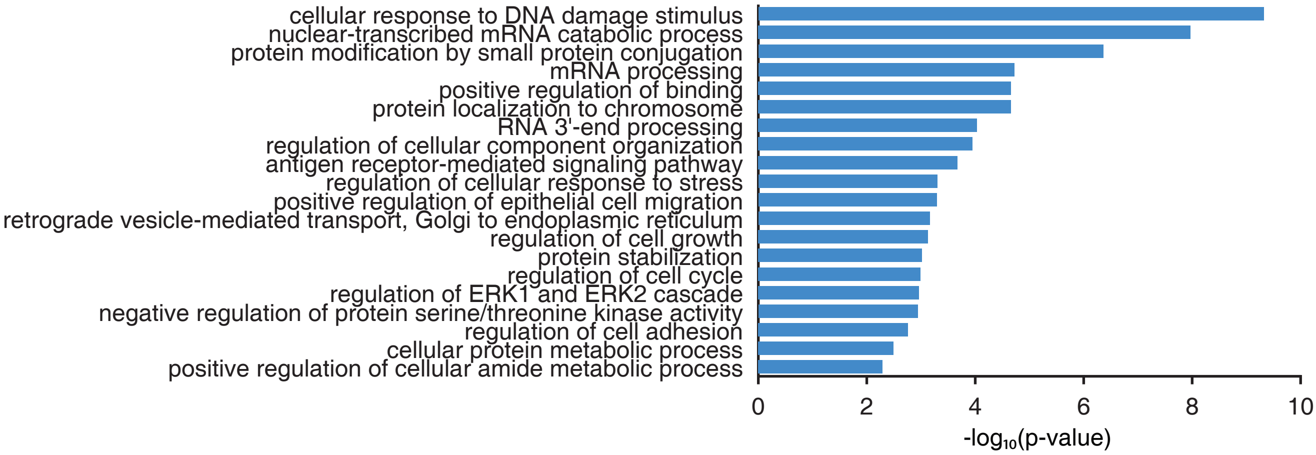

C.

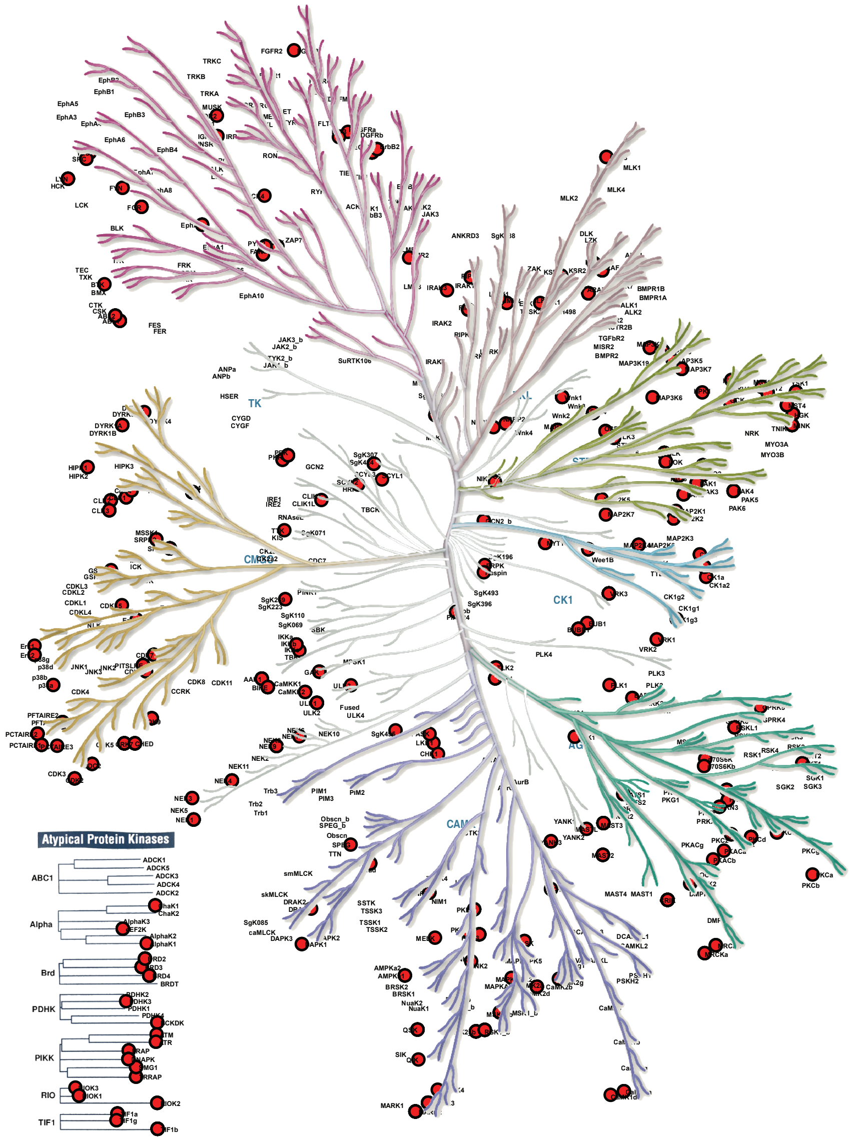

Supplementary Figure S3

A.

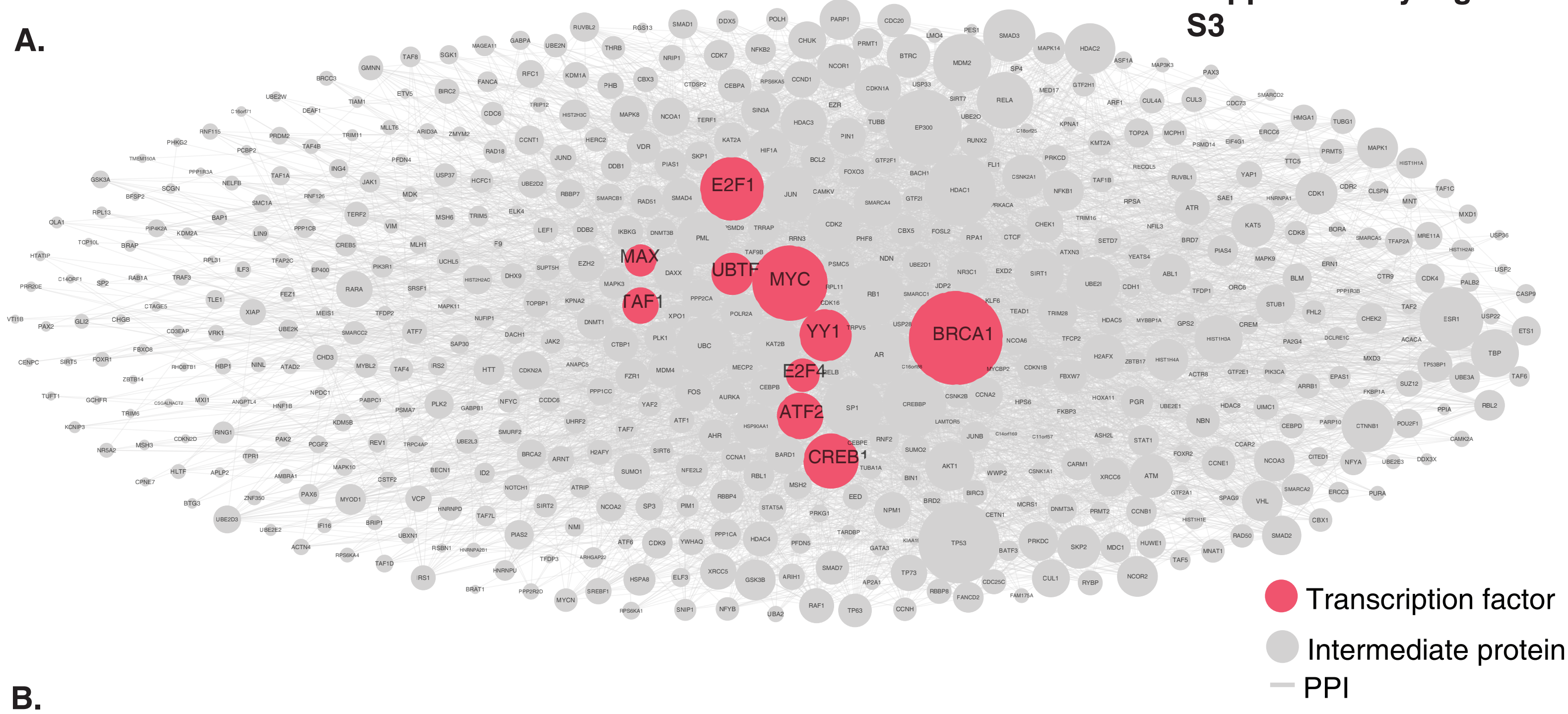

B.

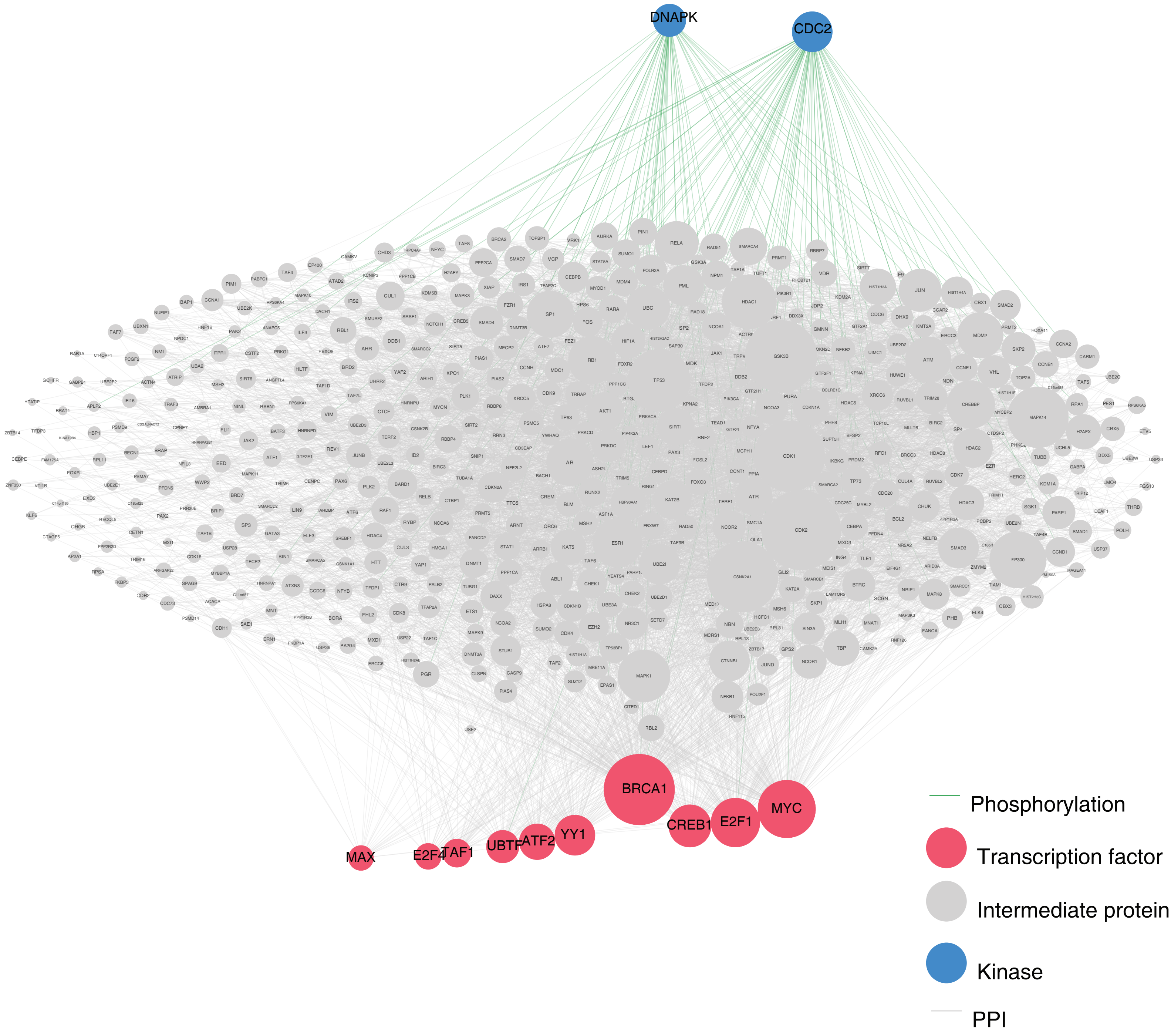

Supplementary Figure S4

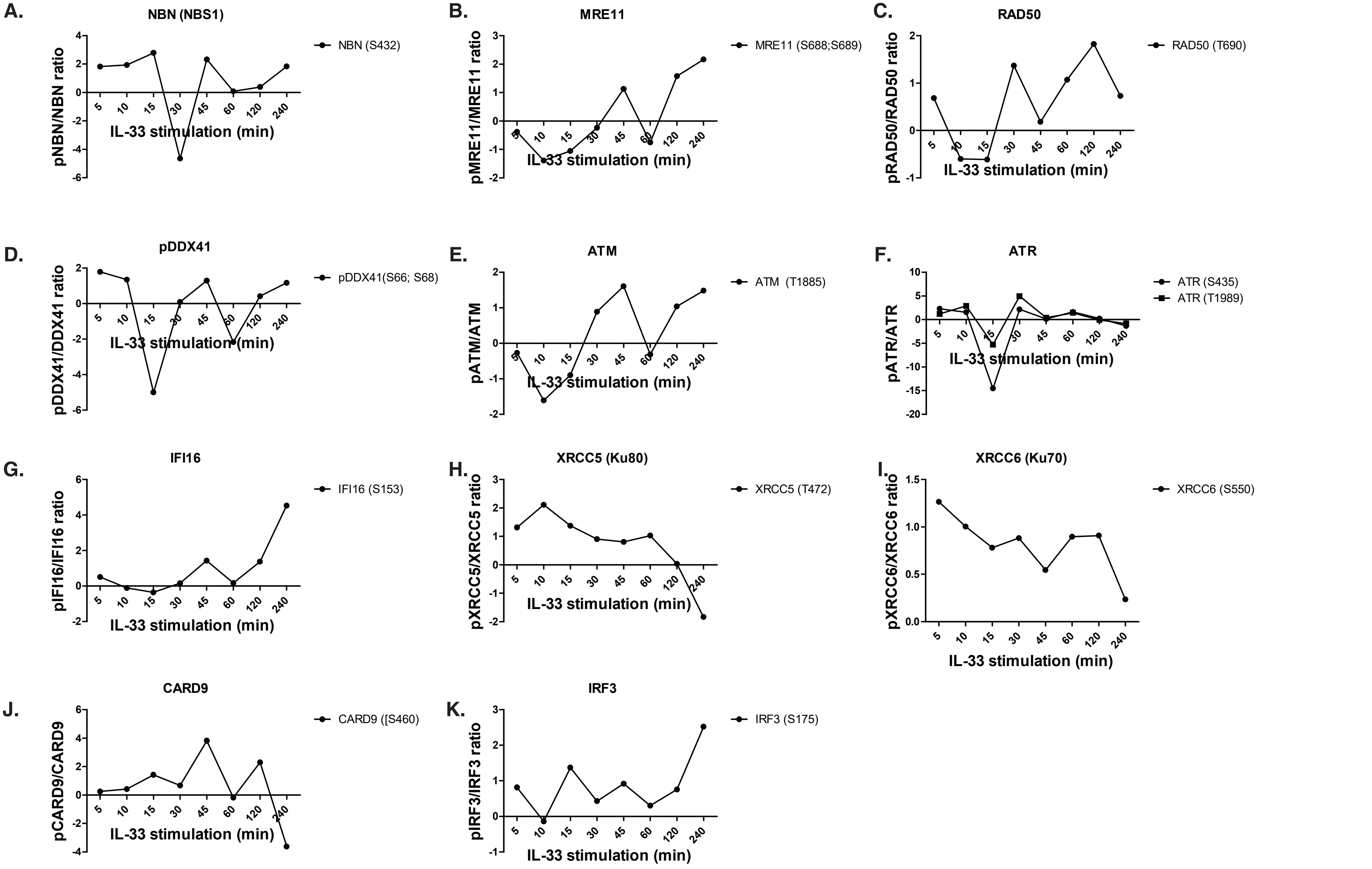

Supplement: Supplementary file 1 [file cells-11-00138-s001.zip › Supplementary_figures.pdf]
